# A critical experimental study of the classical tactile threshold theory

**DOI:** 10.1186/1471-2202-11-76

**Published:** 2010-06-18

**Authors:** Claudio A Perez, José R Donoso, Leonel E Medina

**Affiliations:** 1 Biomedical Engineering Laboratory, Department of Electrical Engineering, and Advanced Mining Technology Center, Universidad de Chile, Casilla 412-3, Av. Tupper 2007, Santiago, Chile; 2 Bernstein Center for Computational Neuroscience, Humboldt-University Berlin, Germany

## Abstract

**Background:**

The tactile sense is being used in a variety of applications involving tactile human-machine interfaces. In a significant number of publications the classical threshold concept plays a central role in modelling and explaining psychophysical experimental results such as in stochastic resonance (SR) phenomena. In SR, noise enhances detection of sub-threshold stimuli and the phenomenon is explained stating that the required amplitude to exceed the sensory threshold barrier can be reached by adding noise to a sub-threshold stimulus. We designed an experiment to test the validity of the classical vibrotactile threshold. Using a second choice experiment, we show that individuals can order sensorial events below the level known as the classical threshold. If the observer's sensorial system is not activated by stimuli below the threshold, then a second choice could not be above the chance level. Nevertheless, our experimental results are above that chance level contradicting the definition of the classical tactile threshold.

**Results:**

We performed a three alternative forced choice detection experiment on 6 subjects asking them first and second choices. In each trial, only one of the intervals contained a stimulus and the others contained only noise. According to the classical threshold assumptions, a correct second choice response corresponds to a guess attempt with a statistical frequency of 50%. Results show an average of 67.35% (STD = 1.41%) for the second choice response that is not explained by the classical threshold definition. Additionally, for low stimulus amplitudes, second choice correct detection is above chance level for any detectability level.

**Conclusions:**

Using a second choice experiment, we show that individuals can order sensorial events below the level known as a classical threshold. If the observer's sensorial system is not activated by stimuli below the threshold, then a second choice could not be above the chance level. Nevertheless, our experimental results are above that chance level. Therefore, if detection exists below the classical threshold level, then the model to explain the SR phenomenon or any other tactile perception phenomena based on the psychophysical classical threshold is not valid. We conclude that a more suitable model of the tactile sensory system is needed.

## Background

The tactile sense is being used in a variety of applications such as human-machine interfaces, telesurgery, virtual reality, robotics and in rehabilitation for the deaf and visually handicapped [1-4]. Different tactile threshold tests are routinely performed with the objective of assessing normal function or diagnose sensory loss in workers exposed to vibration, or in the case of aged or in those patients with disease-related sensory loss such as diabetes, stroke, etc. [5-11]. Vibrotactile excitation is the most widely used form of stimulating the tactile system in human-machine interfaces. In tactile research the threshold concept is widely used and therefore its interpretation is important [1,11-16]. The threshold is defined in classical terms as the minimal quantity of the stimulus that a subject is able to detect [17]. Under classical assumptions, an S-shaped curve called 'psychometric function' (PF) is interpreted as a manifestation of the stochastic nature of the threshold. Therefore, the mean value of the threshold can be obtained from the PF and corresponds to the stimulus level that evokes a 50% of 'yes' responses [11,18,19]. According to the classical threshold concept, in any given trial, sensory events below threshold cannot be detected and are indistinguishable from one another.

Signal detection theory (SDT) emerged as an alternative framework to solve the shortcomings associated with classical threshold theory [19-23]. In the context of a psychophysical experiment, the observer sets a particular value of activation as a criterion upon which to make her/his decision. During a given trial, if the level of activation is above the criterion, the observer chooses 'signal present' and if it is below the criterion the observer chooses 'signal absent' [19,21]. The SDT criterion is just a boundary used to make a decision and it does not impose a sensory limit [24]. Moreover, it can be altered by motivation and bias [18,19,21].

Even though several studies mainly in vision and audition science have supported SDT in opposition to the classical threshold concept [20-23,25,26], there are only a few studies on the validity of SDT applied to the tactile sense [12,27,28]. In particular, the threshold concept, as in the classical or in the SDT interpretation, has not been well discussed in vibrotactile research. In [12] and [27], it was shown that there is no such a threshold as conceived by classical theory and for this limit to be consistent with the data it must be much lower than predicted by classical methods as the middle of the psychometric curve. In [28] it was shown that SDT is a more suitable framework for the study of sensorial processes in tactile perception.

**Figure 1:**
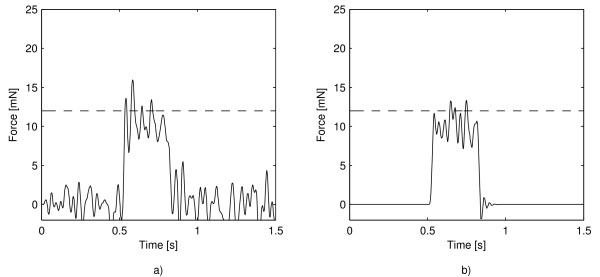
**Example of CESR which cannot be explained by classical theory**. (a)Sample of stimulus with noise in the whole attention interval. (b)Sample of stimulus with noise synchronized in time with the stimulus. The dotted line represents the threshold. In both cases the noise helps the stimulus to surpass the threshold, so that according to classical threshold theory both cases are equally detected. However, experimental results [14] show that detection is better in case b).

In spite of the scientific articles questioning the classical threshold concept, several recent papers use this concept to model and explain psychophysical experimental results in the tactile system [7,8,11,16,29,30], not as a mere definition but as the core of the reasoning. An important example is the phenomenon called stochastic resonance (SR) which occurs when noise is used as a pedestal to improve the detection of sub-threshold stimuli [29,31]. SR psychophysical theory establishes that the amplitude required to exceed the sensory threshold barrier is reached by adding noise to the sub-threshold stimulus [11,32]. If the noise is huge, though, the stimulus is masked and cannot be detected. In these articles, the threshold is considered as a hard barrier below which no sensation can occur so that the interval where noise is not coincident with the stimulus is useless. In a recent paper [14] we showed for the tactile system, a phenomenon of enhanced SR, that we called coincidence-enhanced stochastic resonance (CESR), where improved detection is obtained when the noise is added coincident in time with the stimulus. In this phenomenon a sub-threshold stimulus with noise synchronized in time with the stimulus (Figure 1b), is detected with a higher rate than the same stimulus with noise in the whole attention interval (Figure 1a). According to the classical threshold theory, both cases of sub-threshold stimuli with noise coincident or not coincident in time with the stimulus would yield the same detection [14]. Nevertheless, our results showed enhanced detection when noise is coincident with the stimulus. These results cannot be explained by the classical threshold theory because in both cases it is expected equal detection as the noise acts as a pedestal to help the stimulus to reach the threshold, as shown in Figure 1.

In the present paper we present results of three experiments to show evidence of classical threshold theory failure in the tactile sense from a psychophysical point of view. First we estimated the threshold with a classical psychophysical method. Then we performed a second-choice experiment [22,26] used previously in vision an audition, in which the subject must provide his/her first choice out of three or more alternatives followed by a second choice where he/she thinks the stimulus was presented. Finally we performed a second-choice experiment with low stimulus amplitude.

## Results

### Results experiment 1

The objective of experiment 1 was to locate the 80% correct detection (*X_80_*) on the psychometric function on five young subjects (22-29 years old). The *X_80 _*level produces a significant number of wrong first choices to study the second choice responses. For instance, in experiment 2 we had about 40 samples for each subject with incorrect first choice detection, thus providing 40 samples to test differences in prediction of second choice by classical theory and other models. A three alternative forced choice, 3AFC, procedure was used to sense the observer's response to different stimulus levels. A sequence of 3 intervals was presented to each subject and only one of the intervals, chosen at random, contained the stimulus. The observer indicated in which of the three intervals he/she detected the stimulus. Based on the observer's response, the system automatically updated the level of the stimulus for the next trial by means of a simple weighted up-down adaptive procedure [33]. The vibration amplitude for 80% correct detection averaged 0.64 V (STD = 0.2 V) in the experiment 1, which corresponds to 0.44 mN (SDT = 0.14 mN). This result is summarized in Table 1 that shows the *X*_80 _values for all 5 subjects.

**Table 1:** Results of experiment 1

Subject	***X***_***80***_ [V]
1	0.96

2	0.63

3	0.42

4	0.54

5	0.66

**Mean**	**0.64**

**STD**	**0.20**

Summary of individual's *X*_80 _for 3AFC experiment expressed as voltage level applied to the transducer. It is also shown the mean and the standard deviation.

### Results experiment 2

**Figure 2:**
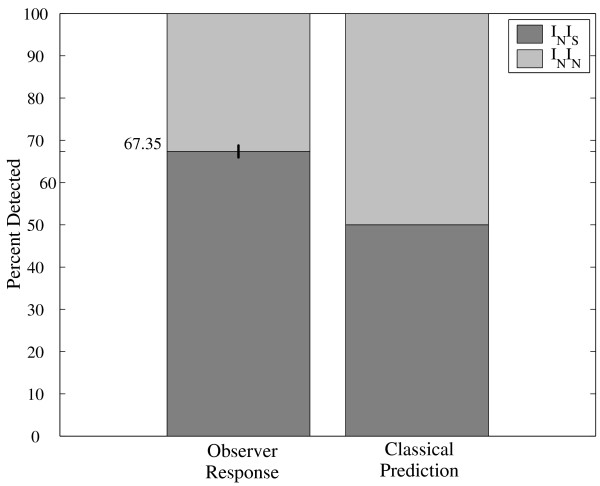
**Results of experiment 2: mean and standard deviation**. Observer Response (left-hand bar) is shown as the mean plus and minus standard deviation for the 5 subjects. It is also shown the Classical Prediction (right-hand bar).

**Figure 3:**
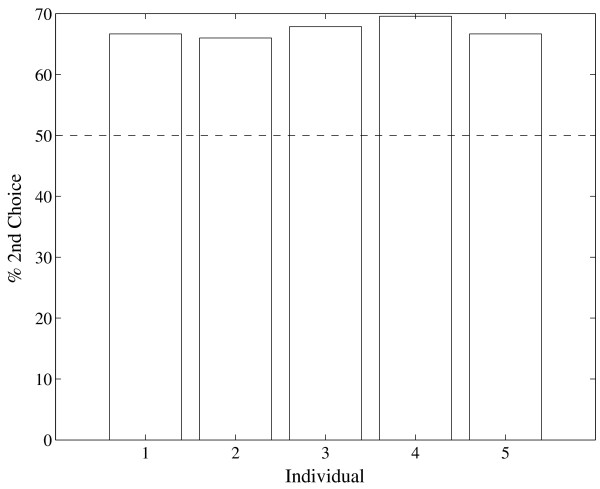
**Results of experiment 2: second choice performance for all subjects**. Second choice correct percentage for the 5 individuals in experiment 2. Segmented line corresponds to chance level, i.e., 50% correct detection.

The objective of this experiment was to analyze the observer's first and second choices under a 3AFC procedure using a constant stimulus level, *X_80_*, obtained from experiment 1. A 3AFC method was performed over the same group of subjects. Only one interval, randomly chosen from the sequence of 3, had a stimulus. The observer had to choose two intervals out of the 3 presented in order of preference considered to be most likely to have contained the stimulus. As expected, correct first choice detection for experiment 2 was about 80%, meaning 77.7% (STD = 4.74%) for the 5 subjects. Let *I_N _*and *I_S _*be intervals containing noise (mechanical stimulus, sensorial neural system, or other type of random disturbance) alone and signal plus noise, respectively. The stacked bars in Figure 2 show 1) the observers' response sequence *I_N_I_S _*and *I_N_I_N_*, i.e., the correct second choice detection and the incorrect first and second choice answer for experiment 2, and 2) the classical prediction of 50% value, i.e., the chance level. The bar labeled "Observer Response" shows a vertical black line centered on the mean (67.35%) and representing plus and minus one standard deviation for the 5 subjects. The percentages are referred to the total number of cases in which the observer did not answer as first choice the signal plus noise interval, i.e., an error in the first choice. The average of 67.35% (STD = 1.41%) is above the chance level predicted by classical threshold hypothesis which is 50% for a 3AFC experiment. This difference is statistically significant according to a binomial test that compares the number of correct second choices for the 5 subjects, 150 out of 223, to the chance level, 111.5 out of 223 (p < 0.001). The results of the second choice for each of the 5 subjects are shown in Figure 3. In this figure, the dotted line shows the predicted value for the classical threshold hypothesis for the 3AFC case. It can be observed that all subjects performed at above-chance levels.

### Results experiment 3

**Figure 4:**
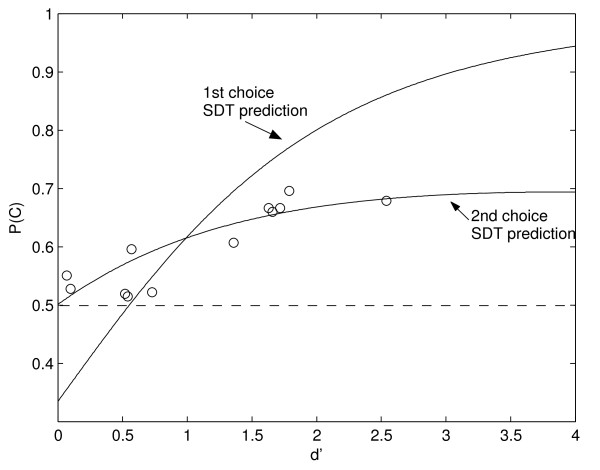
**Results of experiment 3**. It is shown the second choice correct detection against the ***detectability ***estimated from first choice correct detection. It is also shown the predictions of the SDT for both the first and the second choice in the 3AFC experiment. Results of experiment 2 are also plotted and one subject performed two different session of experiment 3, so that 12 points are plotted (5 from experiment 2 and 7 from experiment 3).

The objective of experiment 3 was to analyze the observer's first and second choices under a 3AFC procedure using a low energy stimulus chosen to be just detectable to the individual. Experiment 3 was the same as experiment 2 but using stimulus amplitudes lower than *X_80_*. The first and second choices obtained for the 6 subjects in experiment 3 are summarized in Table 2. In all cases the second choice correct detection is above the chance level, independently of the first choice correct detection. As in experiment 2, the difference was statistically significant according to a binomial test that compares the total number of correct second choices, 536 out of 932, and the chance level predicted by the classical theory with 466 out of 932 (p < 0.001). Figure 4 shows P(C), the percent of correct responses, as a function of *d' *for experiments 2 and 3. The detectability *d' *is a measure of subject's ability to detect the stimulus and it is independent of subject's criterion. In order to estimate *d'*, we used the non-equal variance assumption used in [22,26] with the variance of the signal distribution of the SDT framework varying linearly with the mean of the signal distribution: σ = 0.25 μ + 1 [22]. It can be shown that the SDT model fits very well the data, while the classical model prediction, shown as a horizontal dotted line, cannot explain the scatter of the data.

**Table 2:** Summary of results of experiment 3

Subject	1^st ^choice %	2^nd ^choice %
1	49.5	51.5

2	49.0	52.0

3	36.5	52.8

4	50.5	59.6

5	35.5	55.0

6	69.5	60.7

6-2	55.0	52.2

**Mean**	**49.4**	**54.8**

**STD**	**11.5**	**3.8**

First and second choices for all subjects. Individual 6 performed two sessions.

## Discussion

Classical threshold hypothesis states that the observer is not able to order sensorial events below threshold. Consider experiment 2 in which the observer must pay attention to 3 intervals and let *I_N _*and *I_S _*be intervals containing noise alone (in the context of SDT) and signal plus noise, respectively. Observers have only 3 possible ways of ordering the intervals according to the responses in the 1st and 2nd choice sequence: *I_S_I_N_*, *I_N_I_S _*and *I_N_I_N_*. The last two of them correspond to wrong first choices. Since only one of the 3 intervals contains the stimulus, there are 2 ways of answering *I_N_I_S_*. For example, if the stimulus fell on interval 2, an *I_N_I_S _*answer could be 1-2 or 3-2. Analogously, there are 2 ways to achieve the response *I_N_I_N_*. In our example, the possible intervals are 1-3, 3-1. Therefore, there are 4 possible ways of answering an incorrect first choice, i.e., *I_N_I_S _*and *I_N_I_N _*cases. Classical threshold hypothesis does not consider the possibility that *I_N _*produces a sensorial event higher than the one produced by *I_S_*. Therefore, all 4 possibilities described above have equal probability so that *I_N_I_S _*cases should be 2/4 (i.e., 50%) of the total cases in which the first choice was wrong. On the other hand, SDT does allow observers to order any sensorial event compared to any other even if these are generated under the classical threshold level as in the case of incorrect first choice. According to SDT, when the observer fails in the first choice in the 3AFC, a sensorial event coming from the signal plus noise interval it is more likely to be greater than the other two sensorial events coming from an interval containing only noise. SDT does not provide an exact prediction of a second choice proportion without additional assumptions, but it predicts a second choice to be greater than chance level.

The SDT model does not reject the existence of an abrupt or hard threshold viewed as a limitation of the tactile system to sense extremely weak stimuli. Indeed, it has been shown that several very weak vibrotactile signals of 60 Hz produce detection probabilities which are virtually equivalent when the signals do not exceed 1 micrometer in amplitude [12]. However, it must be emphasized that this limit is much lower than thresholds estimated by classical methods, which are also dependent on observer's criterion. Thus, if the probability of appearance of the stimulus P(S) is set at 0.3, the stimulus value that is detectable 50% of the time equals 1.3 micrometers, while it equals 2.3 micrometers for P(S) of 0.7 [12]. This emphasizes the usual misconception of the threshold concept. Moreover, other threshold models could be hypothesized and could embrace our results and those described above. Indeed, a modification of SDT that includes a low-threshold below which the observer cannot perceive stimuli could be in agreement with the second-choice experiment [26]. Nevertheless, a classical high abrupt threshold is incompatible with our results.

The rejection of the classical high abrupt threshold implies that this concept is not suitable to explain the SR effects observed in psychophysical experiments involving the tactile system. In a recent paper [14], we proposed that in SR this phenomenon could be due to reduction of uncertainty given that noise points to the temporal window where the stimulus is present. In [14], we showed experimental results that agree with the uncertainty hypothesis. However, SR in the tactile system at a physiological level [34] or using EEG recordings [35] is not explained through the classical psychophysical threshold.

## Conclusions

Our findings are in strong contradiction with the classical threshold conception used in psychophysics, just as in [14]. Using a second choice experiment we have shown that individuals can order sensorial events below the level known as a classical threshold. If the observer's sensorial system is not activated by stimuli below the threshold, then a second choice could not be above the chance level. To the contrary, we have shown that a second choice in a 3AFC experiment is better than chance. Our findings are consistent with what was previously shown in the case of visual detection. Importantly, a more suitable model of the tactile sensory system is needed to explain phenomena such as psychophysical SR, design research methods, to interpret experimental results, and for developing prosthetic strategies.

## Methods

### Experiment 1: single psychometric function point estimation

**Figure 5:**
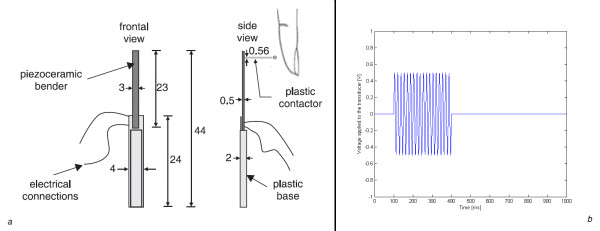
**Experimental setup**. (a) Schematic representation of the piezoelectric stimulator, the plastic base and the plastic contactor. The numbers indicating length, width and height are expressed in millimeters. (b) Voltage waveform applied to the piezoelectric stimulator in each trial, showing the 250 Hz sinusoidal burst (not in scale) within the 1 s presentation interval.

The objective of this initial experiment was to locate a specific point on the psychometric function corresponding to 80% correct detection (*X*_80_), for subsequent testing of each subject. For this purpose a three alternative forced choice, 3AFC, procedure was used to sense the observer's response to different stimulus levels. In this experiment, a sequence of 3 intervals was presented to the subject. Only one of the intervals, chosen at random, contained the stimulus. The task of the observer was to indicate in which of the three intervals he/she detected the stimulus. Based on the observer's response, the system automatically updated the level of the stimulus for the next trial by means of a simple weighted up-down adaptive procedure [33]. In this method, every time the observer made a mistake, the level of the stimulus was increased 4 steps (0.12 V); otherwise the level was decreased 1 step (0.03 V). Starting stimulus level was chosen to be sufficient to be clearly detected by the subject. After about 50 trials a convergence was reached to the expected level *X_80 _*[33], otherwise the experiment was repeated. The method was applied on 5 young healthy subjects (22-29 years old, three males, two females, all right handed), supervised by a researcher to control for attention. In order to avoid fatigue, every test lasted no longer than 20 minutes. Each interval lasted 1 s and subsequent intervals followed immediately after each other without delay. The 1 s interval containing the stimulus was composed of an initial 100 ms gap with no signal followed by a 300 ms burst of 250 Hz sinusoidal waveform and a second 600 ms gap with no stimulus, as shown in Figure 5(b). The 250 Hz frequency was used to excite mainly the Pacinian receptors [15,36]. Table 3 shows the relation between voltage applied to the transducer and displacement with load.

**Table 3:** Relation between voltage amplitudes and displacement of the loaded transducer

Voltage [V]	Displacement [μm]
0.33	0.89

0.39	0.96

0.42	1.03

0.54	1.23

0.63	1.37

0.66	1.44

0.96	1.78

The experiments were approved by the Bioethics Committee, INTA, Universidad de Chile (resolution No.11, June 14, 2006), and the informed consent was obtained from all subjects.

### Experiment 2: first and second choice in the 3AFC method

The objective of this experiment was to analyze the observer's first and second choices under a 3AFC procedure using a constant stimulus level, *X*_80_, obtained from experiment 1. A 3AFC method was performed over the same group of subjects. Only one interval, randomly chosen from the sequence of 3, had a stimulus. Timing characteristics were as described in experiment 1. The task of the observer was to order sensorial events by choosing two intervals out of the 3 presented in order of preference considered to be most likely to have contained the stimulus. Two hundred trials were performed for experiment 2 on each of the 5 subjects. Also the actual stimulus level was recorded. Experiment 2 lasted between 30-40 min for each subject.

### Experiment 3: first and second choice in the 3AFC method at low-energy stimuli

The objective of this experiment was to analyze the observer's first and second choices under a 3AFC procedure using a low energy stimulus chosen to be just detectable to the individual. Experiment 3 was the same as experiment 2 but using stimulus amplitudes lower than *X*_80_. The timing characteristics were also identical to those of experiment 2. Two hundred trials were performed in experiment 3 on six subjects, that is to say, the same group of subjects plus a new one who performed this experiment twice.

### Experimental system

The experiments were performed by stimulating the distal part of the index finger using a plastic circular contactor of 1.5 mm diameter mounted on a bimorph rectangular piezo-electric transducer from Morgan Matroc, with dimensions 23 mm length, 3 mm width and 0.5 mm thickness used previously [13,14] as shown in Figure 5(a). The piezoelectric was mounted in a cantilever manner on a plastic base free to oscillate. This arrangement was mounted on a steel balanced structure to maintain a constant force on the index finger of 0.022 N. The fingers rested on an acrylic cover with a circular perforation of 5 mm diameter to allow the contactor to touch the skin. The index finger was selected for these experiments because it has been used in assistive devices [37] and is the principal area used for tactile exploration because of its high spatial resolution [13,38-40].

The waveform and amplitudes were preprogrammed on a computer using 8 bits per sample. Each sample was sent through the parallel port of the computer to an external D/A converter (DAC0808) and operational amplifier LF353 at 200,000 samples/sec. The waveform was then filtered and amplified by an LM343 power operational amplifier polarized between ± 40 V. The average signal to noise ratio at the transducer was 40.8 dB and there was no artificial noise introduced in excitatory waveform. The time involved in changing from one stimulus amplitude to another was not significant since the waveform was preprogrammed. The waveform and the parameters have been used in previous studies to determine stochastic resonance on the tactile system [14], power consumption [13,41] and two-point spatial resolution [38]. One day prior to the test, all individuals were familiarized with the vibrotactile measurement method, in two 20 min sessions.

## Authors' contributions

CAP contributed in the original research design, particularly in defining the objectives, methodology, discussion and conclusions. LEM contributed with the detailed design of the methods, experimental implementation, experimental data gathering, data analysis and conclusions. JRD contributed in data gathering and analysis. All authors read and approved the final manuscript.
